# First record of gregarine protists (Apicomplexa: Sporozoa) in Asian fungus-growing termite *Macrotermes barneyi* (Blattaria: Termitidae)

**DOI:** 10.1038/s41598-020-79671-7

**Published:** 2021-01-13

**Authors:** Shuo Zhang, Zijia Lin, Qihong Huang, Yulong Shen, Jinfeng Ni

**Affiliations:** State Key Laboratory of Microbial Technology, Microbial Technology Institute, Shandong University, Qingdao, 266237 Shandong China

**Keywords:** Entomology, Parasite biology

## Abstract

*Macrotermes barneyi*, widely distributed in southern China, is the major fungus-growing termite in the subfamily Macrotermitinae. It has no flagellated protists in the guts. Here, we report occurrence of gregarine, a protozoan parasite in the digestive tract of *M. barneyi*. The general morphology and ultrastructure of the gregarine gamonts and syzygies by light micrograph and scanning electron micrograph are presented. SSU rDNA sequence analysis showed that the termite gregarine has the highest identity (90.10%) to that of *Gregarina blattarum* from cockroaches. Phylogenetic analysis based on the SSU rDNA sequences from diverse insect eugregarines indicated that the gregarine from *M. barneyi* is phylogenetically close to *G. blattarus, L. erratica* and *G. tropica* from Gregarinidae and Leidyanidae families, and may represent a novel species. This study expands our knowledge about the diversity of terrestrial eugregarines parasitizing in termites.

## Introduction

Apicomplexan represents a diverse group of unicellular eukaryotes which parasitize the body cavities or the cells of animal. This group include some species pathogenic to human and animals, for example, *Plasmodium* (haemosporidians, causes malaria), and *Eimeria* and *Isospora* (coccodians, cause coccidiosis)^1,2^. Gregarine apicomplexans, closely related to these parasites, are protists that widely inhabit the digestive tracts, fat bodies, Malpighian tubules and reproductive organs of invertebrates from marine, freshwater and terrestrial environments^1,3^. The gregarines are considered to be the earliest diverged within the phylum Apicomplexa, and have several characteristics such as extracellular stage, a monoxenous life-cycle and a prevailing presence in marine hosts. Therefore, the study on gregarines is important for the understanding of the phylogenesis of apicomplexans^1,2^. These protozoan parasites may also good materials for studies on parasite-host coevolution and biological control of insects^3,4^. However, the gregarines have only been poorly explored compared to those much better studied vertebrate pathogens^5,6^.

The gregarines are traditionally divided into Archigregarinorida, Eugregarinorida, and Neogregarinorida based on habitat, morphology and host^1^, and sometimes two groups: Archigregarinorida and Eugregarinorida (with Neogregarinorida incorporating into the Eugregarinorida) are presented^7^. Archigregarines are the ancestral group that exist only in marine environment, and Eugregarines are widely found in marine, freshwater and terrestrial environments^8–10^.

Eugregarines (Eugregarinorida) are recurrent guests in insects, which have been reported in order Blattariam, including cockroaches^11^ and termites^12,13^ and other orders such as Coleoptera, Diptera, Odonata and Orthoptera^11,14–16^. In earlier studies, most of the descriptions were based on morphological traits such as the shape and size of gregarines, which may underestimate the actual diversity. Thus molecular data mainly of 18S rDNA sequences have recently been included to aid taxonomical descriptions^17–19^.

Termites (Blattaria: Termitidae) are eusocial insects and generally divided into lower termites and higher termites based on the presence or absence of flagellated protists in the hindgut of termites^20,21^. The subfamily Macrotermitinae of higher termites, cultivating basidiomycete fungus (*Termitomyces* spp.) in their nests, are known as fungus-growing termites^22^. *Macrotermes barneyi* is one of the major fungus-growing termites, widely distributed in southern China^23^. So far, no gregarine has been reported in Asian termites, as far as we know. In this study, we report a novel occurrence of gregarines in the digestive tract of *M. barneyi,* the morphology at the common life-cycle stages and the first SSU rDNA sequence from termite gregarines.

## Results

### Presence of gregarines in the gut of *M. barneyi*

**Figure 1:**
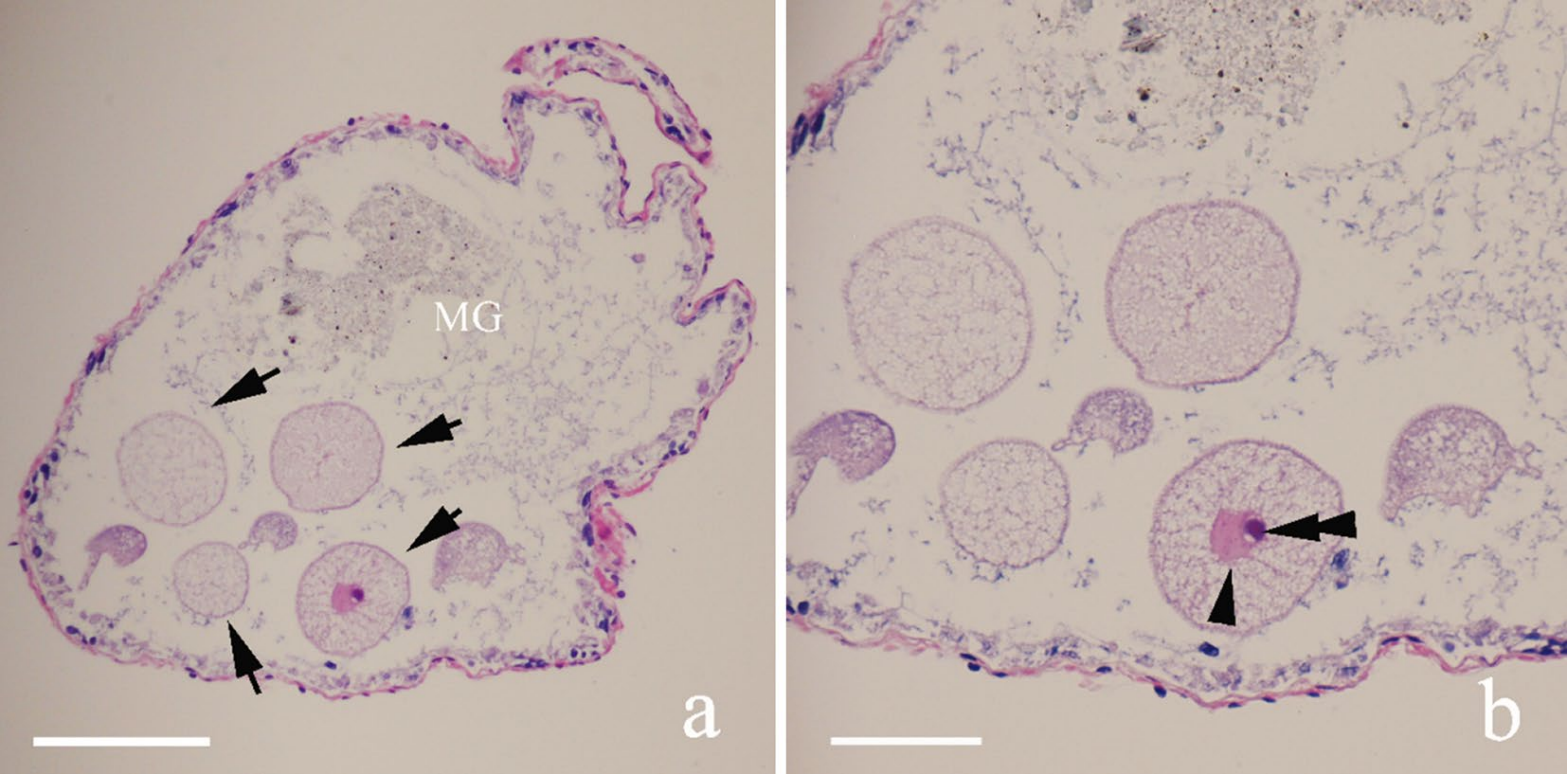
Transversal histological section of the midgut (MG) of *Macrotermes barneyi* stained by HE. (**a**) Four complete section of gregarines (arrowed) shown in the midgut of the infected host. (**b**) Higher magnification of **a** showing the nucleus (arrowhead) and nucleolus (double arrowhead). Scale bars (**a**) 100 μm; (**b**) 50 μm.

The gregarines were firstly found in the midgut of termite worker including both minor and major workers of *M. barneyi.* The abundance of gregarines was higher in the minor worker termites, ranging from dozens to hundreds. In one paraffin section of the intestinal part of the termite worker (Fig. 1), four complete cross sections of the gregarine were shown (Fig. 1a, arrow). The nucleus (arrowhead) and nucleolus (double arrowhead) were clearly visible in one cross section (Fig. 1b). None of gregarine was observed in soldier termites. The percentage of termite worker with infected gregarines was 21.45% (Table1).

**Table 1:** The termite origin and infection rates of gregarine in *Macrotermes barneyi.*

Termite species	Location	Caste	Termites with dissection	^#^Termites with gregarines	Infection rates %
*Macrotermes barneyi*	Zhongshan, Guangdong Collecting: 113° 27″ E, 22° 49″ N Feeding: 113° 31″ E, 22° 48″ N	Workers	303	65	21.45
		Soldiers	29	0	0

^#^Only gregarines observed in the midgut were counted.

### Morphology of the gregarines

**Figure 2:**
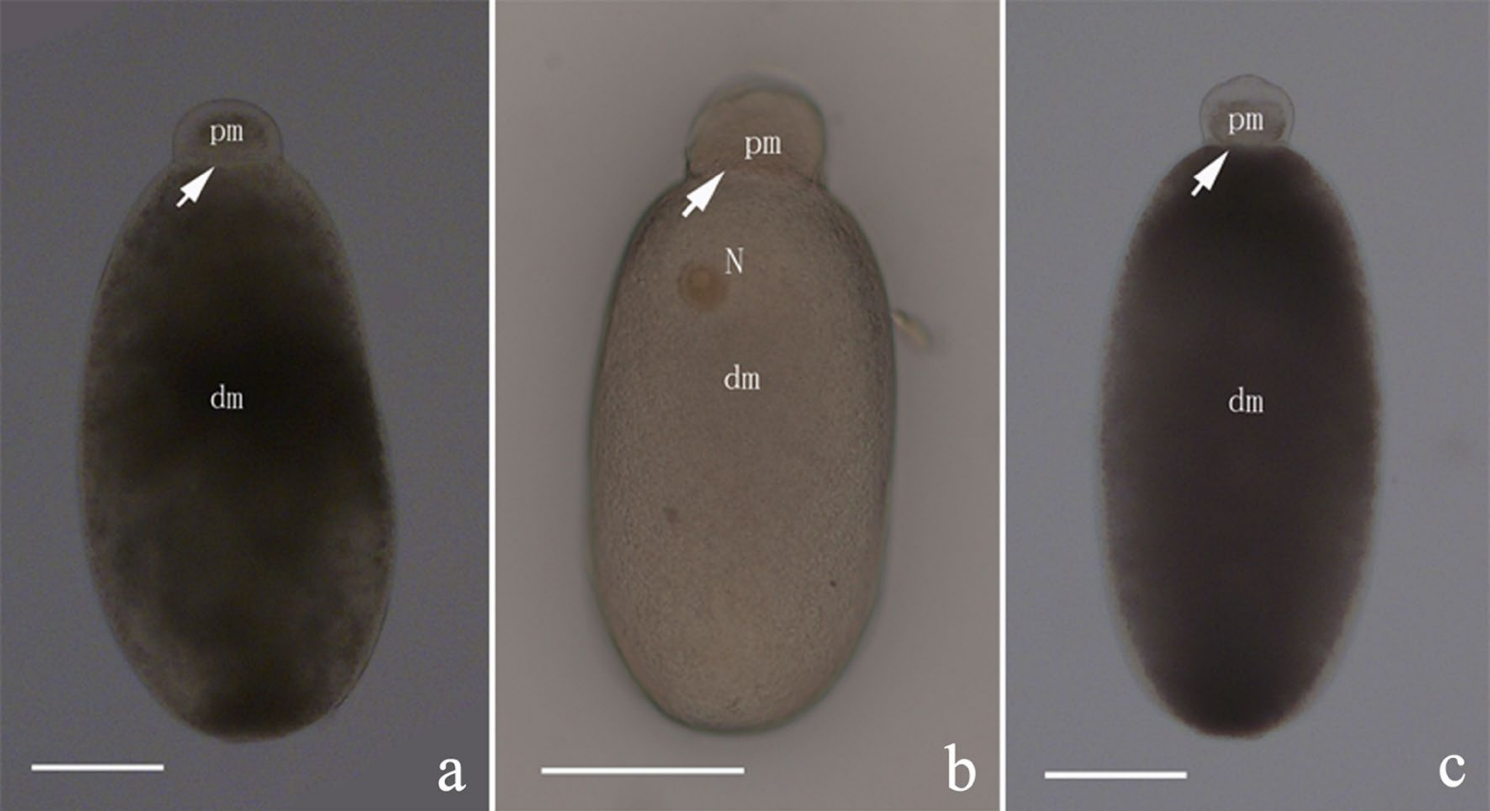
Morphology of gamonts observed in different termite individuals by light microscope. The gamonts were isolated from the midgut of termite workers. In (**a**,**c**) the samples were observed under differential interference contrast (DIC) light microscope, while in (**b**) the sample was observed under bright field microscope. The cell contains two compartments: protomerite (pm) and deutomerite (dm). The nucleus (N) was visible in the anterior part of the dm (**b**), and the septum (arrow) separated the pm and dm. Scale bars (**a**–**c**) 100 μm.

**Figure 3:**
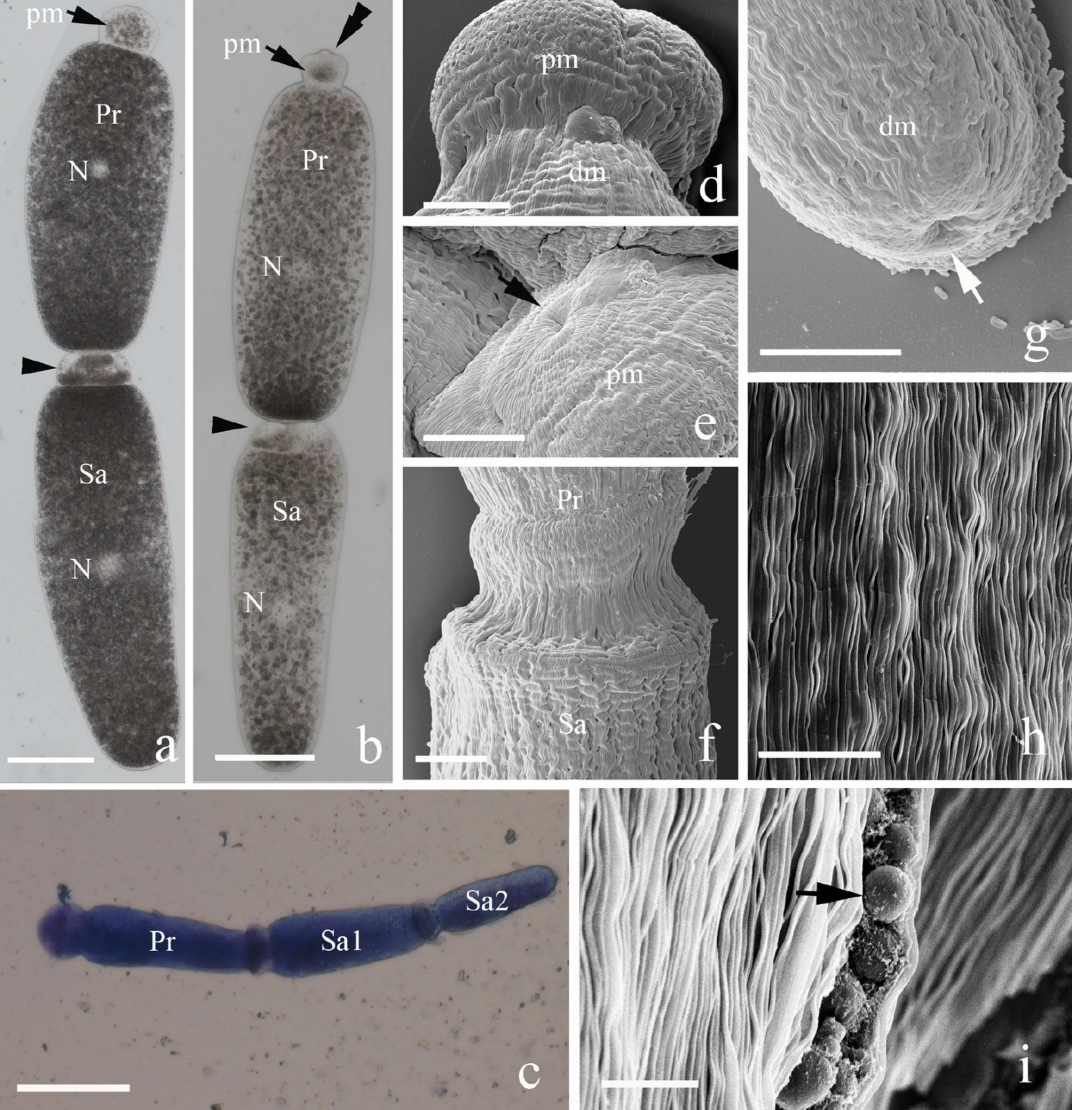
Light micrograph (LM) and scanning electron micrographs (SEM) showing the morphology and surface ultrastructure of the syzygies from the midguts of *M. barneyi*. (**a**,**b**) LM of syzygies. The syzygy contains two parts: primite (Pr) and Satellite (Sa). The nucleus (N) is visible in the middle of each individuals. The top structures in Pr (double arrowhead) and the connection (arrowhead) between the Pr and Sa were different between (**a**,**b**). (**c**) LM of a syzygy with three parts dyed by Meilan dye solution. (**d**–**g**) SEM showing the partial structure of syzygy; (**d**) the connecting part between protomerite (pm) and deutomerite (dm) in Pr.; (**e**) showing a recessed area (arrow) in the center of the top of the pm in (**a**); (**f**) higher magnification of the connecting part between Pr and Sa; (**g**) SEM of the posterior end of the Sa. (**h**,**i**) SEM showing the surface structure of syzygy; the ball-like structure was observed from the ruptured surface in (**h**). Scale bars (**a**,**b**) 100 μm; (**c**) 50 μm; (**d**–**g**) 10 μm; (**h**,**i**) 2.5 μm.

Most of gregarines observed in the midgut were gamonts (Fig. 2) and syzygies (Fig. 3a,b). The gregarines were in brownish yellow or brownish black color. The gamonts contained a protomerite (pm) and deutomerite (dm) that can be differentiated by size and the presence of the nucleus. The gamonts were divided by an always present septum (Fig. 2, arrow). The front part pm was in flat ellipsoid, with the lengths ranging from 26.8 to 65.1 μm, and the widths ranging from 36.7 to 86.4 μm. The dm was long ellipsoid with a length from 182.1 to 377.2 μm and widths from 55.8 to 172.2 μm, respectively (Table 2). The nucleus (N) was located in the upper middle portion of the posterior segment (Fig. 2b).

**Table 2:** Morphometric data of gregarines from this study.

Characters	Mean (μm)	SD (μm)	Min (μm)	Max (μm)	Numbers
**Gamonts**					
Protomerite length	42.8	11.7	26.8	65.1	10
Protomerite width	55.3	15.6	36.7	86.4	10
Deuteromerite length	321.4	53.8	182.1	377.2	10
Deuteromerite width	120.4	34.0	55.8	172.2	10
**Syzygies**					
Prime length	348.7	78.0	202.8	563.6	20
Satellite length	325.6	84.9	119.4	472.7	20
Total length	674.3	149.4	322.2	945.4	20
Prime width	112.2	28.7	53.2	171.8	20
Satellite width	105.4	24.3	59.9	165.7	20
Total width	217.6	45.3	130.9	311.1	20
**Gametocysts**					
Diameter	135.9	31.3	82.3	197.2	10

*Max* maximum, *Min* minimum, *SD* standard deviation.

**Figure 4:**
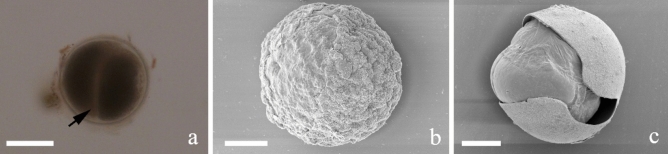
Differential interference contrast (DIC) light micrograph and scanning electron micrographs (SEM) showing the general morphology of the gametocyst from the termite hindgut. (**a**) DIC light micrograph of gametocyst showing initial stage of gametocyst formation in which individual gamonts associate (arrow) to form a spherical structure. (**b**) SEM of gametocyst showing the surface ultrastructure. (**c**) A mechanically ruptured gametocyst. Scale bars (**a**) 200 μm; (**b**) 50 μm; (**c**) 100 μm.

The syzygies consisted of two or three gamont connected caudofrontally (Fig. 3a–c). The majority of syzygies were composed of two cells (Fig. 3a,b). The first gamont of the syzygies is known as primite (Pr) and the remaining cells are called satellites (Sa). The top of pm was presented in different shapes: a smooth anterior tip (Fig. 3a) and a papillary protrusion (Fig. 3b). The SEM structure between pm and dm in Pr was shown in Fig. 3d. The high magnification of the apical part of the pm (Fig. 3e), the connecting section between Pr and Sa (Fig. 3f) and the end recessed area of Sa (arrow, Fig. 3g) were also shown. The longitudinal surface of the observed gamonts and syzygies were shown in Fig. 3h. From the split of the surface, ball-like structures (about 1.2–2 μm in diameter) were observed (Fig. 3i). The length and width of two-cell formed syzygies ranged from 322.2 to 945.4 μm and 130.9 to 311.1 μm, respectively. The three-cell formed syzygies was about 236.8 μm in length and 69.9 μm in width, and the size was significantly smaller than that of two-cell formed syzygies. Gametocysts were found in the hindgut of *M. barneyi* and feces (Fig. 4). The spherical gametocysts contained two encysted gamonts with a transparent outer surface (Fig. 4a). The surface ultrastructure of gametocyst by SEM was shown in Fig. 4b,c. The average mean diameter of gametocysts was 135.9 μm (Table 2).

### SSU rDNA sequence and phylogenetic tree analysis

The SSU rDNA sequence (MT126033) was obtained by sequencing of the PCR fragment amplified from the termite gregarine parasite. It has a length of 1778 bp and GC content of 48.73%. The majority of reported SSU rDNA sequences are among 1500–1800 bp except for two short sequences (1200 bp) from *Gregarina chortiocetes* (L31841.1) and *G. caledia* (L31799.1). Homology analysis showed that the SSU rDNA sequence of gregarine from termite *M. barneyi* shares approximately 89–90% identity to the terrestrial eugregarines from cockroach, beetle and cricket. Among three SSU rDNA sequences from insect gregarines, the sequence from the termite *M. barneyi* gregarine (MT126033) has the highest identity (90.10%) to that of gregarine from cockroach (FJ459741).

**Figure 5:**
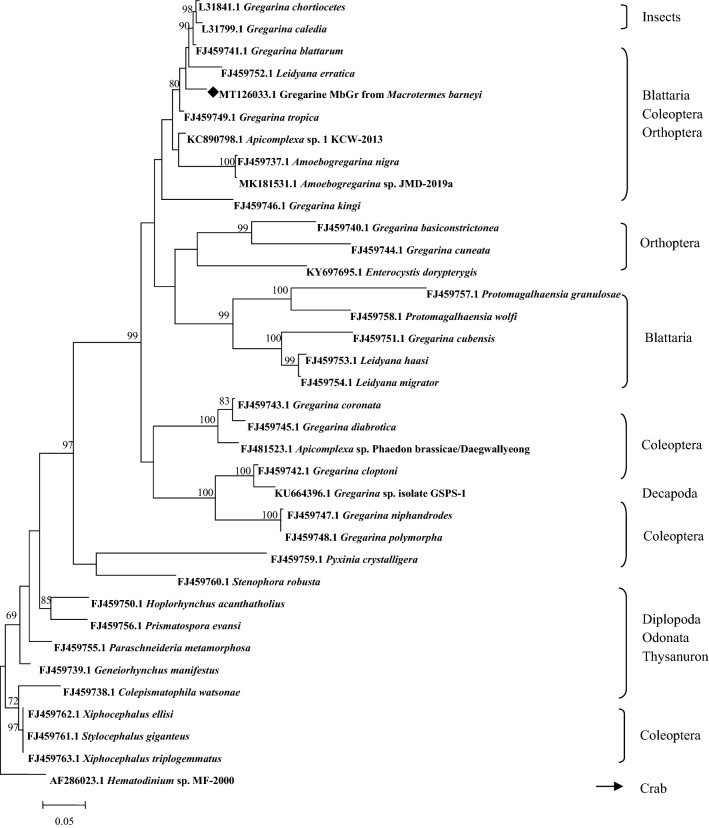
Molecular phylogenetic analysis using SSU rDNA sequences from termite gregarine (black marked) and other eugregarines from various insects. The order of insect hosts was listed on the right of the tree. The tree was constructed by Maximum Likelihood method, and evolutionary analyses were conducted in MEGA7. The dinoflagellates sequence from crab was used as outgroup. The numbers on the nodes indicate the bootstrap values of higher than 60%.

A phylogenetic tree was constructed using the SSU rDNA sequences from insect eugregarines (Eugregarinorida). These gregarines belong to the following families: Gregarinidae, Leidyanidae, Hirmocystidae, Stylocephalidae, Actinocephalidae, Stenophoridae and Enterocystidae. The insect hosts include the Orders Blattaria, Coleoptera, Dermaptera, Diptera, Odonata, Orthoptera, Odonata and Thysanuronb. The individual nodes of the tree were overall well supported for the analysis, but major clades were not well resolved (Fig. 5). The eugregarines from insects formed several clades, and the termite gregarine (MT126033.1) was positioned within a clade that included *Gregarina blattarus*, *Leidyana erratica* and *G. tropica*. The crab parasite *Hematodinium* sp (AF286023.1), designated as the outgroup, was on separate node.

## Discussion

This study describes the occurrence of gregarine protozoa in the intestine of the fungus-growing termite *M. barneyi* in China. The location, morphology and ultrastructure of gregarines were shown by histological section and LM/SEM. Further, the SSU rDNA sequence from termite gregarines and its molecular phylogenetic analysis were presented.

There are very few literatures about gregarine from termites^12,13^. A gregarine (Apicomplexa: Neogregarinida) reported in lower termite *Coptotermes gestroi*, which had a lemon-shaped spores, was suggested to belong to the family Lipotrophidae. The gregarines cysts from workers of *C. gestroi* was ovoid in shape and was about 80–150 μm × 60–110 μm. The size of gametocysts isolated from *M. barneyi* was 82.3–197.2 μm, similar to the above value. According to the book by Desportes^16^, gregarine species found from different termite hosts, belong to the following families: Actinocephalidae, Gregarinidae, Hirmocystidae, Kofoidinae and Sphaerocystidae^16^. These gregarines were recorded by line drawing, and were mainly found in termites distributed in Italy, USA and India. There were not much morphometric data of gregarines available from these termites. The syzygies of two gamont-connected was 400 μm for gregarine of *Pleatospora termitis* (the host is *Macrotermes estherae*), and the total length of syzygies gamonts of *Kofoidina ovata* from termite *Zootermopsis nevadensis* in association of 2–14 gamonts was 636 μm^16^. The size of gregarines from different termite species are variable. In this study we have presented the detailed morphometric data of common life stages (gamont, syzygy and gametocyst), which would be useful for further characterization of gregarines.

Termites are known as eusocial insects, which include the reproductive, worker and soldier termites. The gregarine was initially found in the worker of *M. barneyi*, then the presence of gregarine in the digestive tract of king, queen and soldier termites were examined, and no gregarine was found in these castes of *M. barneyi*. Similar phenomenon was reported in other termite workers^13^ and wasps workers^24^. Workers and soldiers from field colonies of lower termite *C. gestroi* were checked for the presence of gregarine, the gregarine cyst was found only in worker termites^13^.The gregarines seem to be specific to workers, the reason might be related to the roles of termites. The worker termites mainly collect foods and transfer them to the nest, which increase their exposure to the parasite oocysts. An alternative explanation may be that gregarine infection is related to host’s natural microbiota. The forage termite workers have a more diverse microbiome than other termite castes^1,25^, which may make workers more suitable to harbor gregarines. Of course, further studies on host–microbe interactions should be explored.

It is noted that infection rate (frequency of the infected individuals), here in *M. barneyi*, was about 21%, which is higher than that reported in termites *C. gestroi* (0.6–3.3%)^13^ and *Odontotermes* sp (5%)^12^. The rate of gregarine infection varied with different host species, and even changes with different seasons in same species, which could reach the highest 35% in workers of *Polybia* species, neotropical swarm-founding wasps^24^. By now, the effect of gregarines on the host has not been clarified, no effects or the negative effects ranging from high mortalities to negligible effects have been reported in all cases^1,26–29^. This seems to depend on the quantity and gregarine species as well as environment of the host’s habitat^1^.

The invertebrate hosts are usually infected with gregarines by swallowing mature oocysts, which are released into the environment through feces and the gregarines are often found in the digestive tract and body cavity of the insect^11^. In the current study, three life-cycle stages of gregarines such as gamont, syzygy and gametocyst have been obviously observed in midgut, hindgut and/or fece of *M. barneyi*, and other stages of the gregarine have not been found. It was difficult to maintain fungus-growing termite *M. barneyi* alive in the laboratory for a long time. We noted that gregarine has the papilla like structure at the top of the cell (Figs. 2c, 3b), suggesting that they are probably trophozoites. However, more morphological data are needed to give an accurate taxonomic identification of the gregarine. The majority of gregarines observed are two-cell type of syzygies and we observed the three-cell type of syzygies once. Multiple or bi-association syzygies was reported in Hirmocystidae family such as type species *Kofoidina ovata*^16^, and it was an aseptate species found in the gut of *Z. nevadensis*, while the gregarine observed in *M. barneyi* is septate gregarine. The current data suggested that this gregarine found in *M. barneyi* might be a novel species. The species name has not been determined due to incomplete morphological structure and limited molecular sequence data available from termite gregarines. Although the major clade in phylogenetic analysis is not well resolved, overall tree topology is generally consistent to the reported analysis^7,14,17^, which indicates that the gregarine from *M. barneyi* is phylogenetically close to *G. blattarus, L. erratica and G. tropica* from families of Gregarinidae and Leidyanidae, and belongs to Gregarinidae group I^14^.

In summary, we report the presence of the gregarine in a fungus-growing termite *M. barneyi* for the first time. The current study expands the understanding of distribution and diversity of terrestrial eugregarines in insects, and enriches our knowledge on termite parasites, which will contribute to the further gregarines study and phylogenetic evolution research.

## Materials and methods

### Termite collection, feeding and identification

*Macrotermes barneyi* colony harboring all castes and fungus combs was collected from Zhongshan, Guangdong Province in southern area of China (113° 27″ E, 22° 48″ N). The colony was carefully wrapped and brought to the laboratory. After arriving the lab, the dead termite and broken combs were removed, and the complete fungus combs with healthy termites were put into a customized plastic container, which was kept in incubator at 27°and 85% humidity . The termite species was identified by the morphology of the soldier and mitochondrial COII gene sequencing^30^.

### Termite dissection and isolation of the gregarines

Worker and soldier termites were rinsed in distilled water, 75% ethanol and distilled water in turn, then the termites were dissected and the whole guts were removed to the sterilized plates. The guts were separated into foregut, midgut and hindgut under Olympus SZ2-ILST dissection microscope (Olympus Corporation, Tokyo, Japan). The gregarines were checked in each region of the digestive tract and were isolated carefully using pipette for observation or stored at − 20° for later DNA extraction.

### Paraffin section preparation and observation

After dissection, the midguts were transferred into 4% paraformaldehyde PB buffer and fixed for 24 h. The samples were prepared for paraffin sectioning and stained with HE (hematoxylin–eosin) according to protocol providing by Wuhan Sevier Biotechnology Co., Ltd. The obtained slides were observed and photographed with a Nikon Eclipse80i bright-field microscope (Nikon Corporation, Tokyo, Japan) equipped with a Nikon DS_Ri1 (Nikon Corporation, Tokyo, Japan) camera.

### Light microscope observation

Live observations of the gregarines were performed with a Nikon Eclipse80i differential interphase contrast (DIC), and bright field microscope (Nikon Corporation, Tokyo, Japan) equipped with a Nikon DS_Ri1 camera for microphotographic records of the cells. Optical micrographs were arranged using Adobe Photoshop CS5 (Adobe Systems Incorporated, San Jose, CA, USA). The width and length of each compartment of gregarines were measured at different stage, mainly gamonts and syzygies.

### Scanning electron microscope observation

For scanning electron microscope (SEM), The gregarines were fixed in 2.5% glutaraldehyde for 3 h, after which the cells were transferred to PBS buffer and washed three times to wash off the fixing solution, dehydrated with 10%, 30%, 50%, 70%, and 90% (v/v) ethanol for 15 min each time, and then in anhydrous ethanol for 15 min twice. The solution containing the gregarines was dropped in the center of the slide, and the slide carrying the sample was placed in a Leica EM CPD300 critical point dryer for drying. The slide was then adhered to the sample stage with conductive tape and gold plated for 240 s using a Cressington 108 ion sputtering device. SEM stubs were observed and photographed under a Quanta 250 FEG scanning electron microscope (Thermo Fisher Scientific, Massachusetts, USA), and the photos were edited with Adobe Photoshop CS5.

### DNA extraction and sequencing

Approximately 50 gamonts or syzygies were taken and pooled from *M. barneyi* for DNA extraction. After washing with PBS buffer three times, the genomic DNA of gregarines was extracted following the instruction provided by the TIANamp Mico DNA Kit (Tiangen Biotech Co., Ltd., Beijing, China). DNA concentration and quality were detected using a Neno-200 micronucleic acid analyzer (Allsheng Instruments Co., Ltd, Hangzhou, China). For PCR amplification of the SSU rDNA, two universal primers: F1 (5′-GCGCTACCTGGTTGATCCTGCC-3′) and R1 (5′-GATCCTTCTGCAGGTTCACCTAC-3′) were used^2^. The total volume of the amplification reaction mixture is 50 μL, which contained 25 μL of 2X M5 Taq HiFi PCR MIX (Mei5 Biotechnology Co., Ltd., Beijing, China), 1 μL each of upstream and downstream primers (10 μM), 1 μL of template DNA (about 45 ng) and ddH_2_O 22 μL. The PCR was performed in a TaKaRa TP600 thermal cycler (Takara Bio Inc., Dalian, China) using the following procedure: initial denaturation at 94 °C for 5 min; followed by denaturation at 94 °C for 45 s, annealing at 55 °C for 45 s, 72 °C for 2 min, and final extension at 72 °C for 10 min. The amplified fragment was ligated into pMD19-T vector (Takara) and the recombinant plasmid was sequenced by Shanghai Shengong Company. The sequence was analyzed using BLAST (http://blast.ncbi.nlm.nih.gov).

### Molecular phylogenetic analysis

SSU rDNA sequence from *M. barneyi* gregarines (MbGr), deposited in GenBank under accession number MT126033.1, was aligned with other 35 apicomplexan sequences mainly from insects (min coverage > 52%, identity > 77.28%), and the dinoflagellate *Hematodinium* sp. (AF286023) was used as an outgroup (Supplementary Table S1). The alignment was performed on MEGA 7 using MUSCLE algorithm with default parameters^31^. After alignment, the obvious gaps were deleted. The evolutionary history was inferred by using the Maximum Likelihood method based on the Tamura-Nei model^32^. The tree with the highest log likelihood (− 9754.60) is shown. The percentage of trees in which the associated taxa clustered together is shown next to the branches. Initial tree(s) for the heuristic search were obtained automatically by applying Neighbor-Join and BioNJ algorithms to a matrix of pairwise distances estimated using the Maximum Composite Likelihood (MCL) approach, and then selecting the topology with superior log likelihood value. The tree is drawn to scale, with branch lengths measured in the number of substitutions per site. A total of 36 nucleotide sequences were used in the analysis. All positions containing gaps and missing data were eliminated.

### General parasitological observations

Both worker and soldier termites were subjected to dissection and the presence of parasites inside the digestive tract were registered. The number of termites and gregarines were counted to calculate the general prevalence and intensity. The infection rate was presented as the percentage of the number of hosts infected at least one parasite cell^13^.

## Supplementary Information

Supplementary Information
