# Supplementary material for: First record of gregarine protists (Apicomplexa: Sporozoa) in Asian fungus-growing termite Macrotermes barneyi (Blattaria: Termitidae)
Source: Sci Rep. 2021 Jan 13;11:989. doi: 10.1038/s41598-020-79671-7 (PMC7806973; doi:10.1038/s41598-020-79671-7)
Supplement: Supplementary file 1 — Supplementary Information [file 41598_2020_79671_MOESM1_ESM.docx]

**First record of gregarine protists (Apicomplexa:Sporozoa) in Asian fungus-growing termite *Macrotermes barneyi* (Blattaria:Termitidae)**

**Shuo Zhang^1^, Zijia Lin^1^, Qihong Huang, Yulong Shen* & Jinfeng Ni***

State Key Laboratory of Microbial Technology, Microbial Technology Institute, Shandong University, Qingdao, Shandong, China, 266237.

*Correspondence: [jinfgni@sdu.edu.cn](mailto:jinfgni@sdu.edu.cn) or [yulgshen@sdu.edu.cn](mailto:yulgshen@sdu.edu.cn)

^1^ These authors contributed equally to this work.

| **Table S1 List of SSU ribosomal RNA gene sequences used in phylogenetic analysis.** | | | | | | |  |
| --- | --- | --- | --- | --- | --- | --- | --- |
| **Identity** | **GenBank number** | **Length (bp)** | **Gregarine species** | **Order** | **Host** | **Order** | |
| 89.14% | L31841.1 | 1210 | *Gregarina chortiocetes* | Eugregarinorida | Insects | Unknown | |
| 88.87% | L31799.1 | 1210 | *Gregarina caledia* | Eugregarinorida | Insects | Unknown | |
| 90.10% | FJ459741 | 1702 | *Gregarina blattarum* | Eugregarinorida | *Blattella germanica* | Blattaria | |
| 89.43% | FJ459752 | 1623 | *Leidyana erratica* | Eugregarinorida | *Gryllus pennsylvanicus* | Orthoptera | |
| 100% | MT126033.1 | 1778 | Gregarine MbGr | Eugregarinorida | *Macrotermes barneyi* | Blattaria | |
| 90.34% | FJ459749 | 1620 | *Gregarina tropica* | Eugregarinorida | *Vostox brunneipennis* | Dermaptera | |
| 91.33% | KC890798.1 | 1780 | *Apicomplexa sp.* 1 KCW-2013 | Unknown | Unknown (Marine gregarine) | Unknown | |
| 85.88% | FJ459737.1 | 1677 | *Amoebogregarina nigra* | Eugregarinorida | *Melanoplus differentialis* | Orthoptera | |
| 87.43% | MK181531.1 | 1764 | *Amoebogregarina taeniopoda* | Eugregarinorida | *Taeniopoda centurio* | Orthoptera | |
| 85.61% | FJ459746 | 1631 | *Gregarina kingi* | Eugregarinorida | *Gryllus pennsylvanicus* | Orthoptera | |
| 79.92% | FJ459740 | 1572 | *Gregarina basiconstrictonea* | Eugregarinorida | *Tribolium castaneum* | Coleoptera | |
| 77.81% | FJ459744 | 1614 | *Gregarina cuneata* | Eugregarinorida | *Tenebrio molitor* | Coleoptera | |
| 80.88% | KY697695 | 1722 | *Enterocystis dorypterygis* | Eugregarinorida | *Dorypteryx domestica* | Psocoptera | |
| 75.78% | FJ459757 | 1601 | *Protomagalhaensia granulosae* | Eugregarinorida | *Blaberus discoidalis* | Blattaria | |
| 79.18% | FJ459758 | 1632 | *Protomagalhaensia wolfi* | Eugregarinorida | *Nauphoeta cinerea* | Blattaria | |
| 78.16% | FJ459751 | 1617 | *Gregarina cubensis* | Eugregarinorida | *Gromphadorhina portentosa* | Blattaria | |
| 82.88% | FJ459753 | 1601 | *Leidyana haasi* | Eugregarinorida | *Nauphoeta cinerea* | Blattaria | |
| 80.99% | FJ459754 | 1670 | *Leidyana migrator* | Eugregarinorida | *Gromphadorhina portentosa* | Blattaria | |
| 80.76% | FJ459743 | 1626 | *Gregarina coronata* | Eugregarinorida | *Diabrotica undecimpunctata* | Coleoptera | |
| 80.84% | FJ459745 | 1547 | *Gregarina diabrotica* | Eugregarinorida | *Acalymma vitattum* | Coleoptera | |
| 81.52% | FJ481523.1 | 1727 | *Apicomplexa sp.* | Unknown | *Phaedon brassicae* | Coleoptera | |
| **Identity** | **GenBank number** | **Length (bp)** | **Gregarine species** | **Order** | **Host** | **Order** | |
| 78.79% | FJ459742 | 1678 | *Gregarina cloptoni* | Eugregarinorida | *Tribolium freemani* | Coleoptera | |
| 79.52% | KU664396 | 1682 | *Gregarina sp. isolate* GSPS-1 | Eugregarinorida | *Atyaephyra desmarestii* | Decapoda | |
| 80.56% | FJ459747 | 1593 | *Gregarina niphandrodes* | Eugregarinorida | *Tenebrio molitor* | Coleoptera | |
| 80.56% | FJ459748 | 1648 | *Gregarina polymorpha* | Eugregarinorida | *Tenebrio molitor* | Coleoptera | |
| 77.28% | FJ459759 | 1610 | *Pyxinia crystalligera* | Eugregarinorida | *Dermestes maculata* | Coleoptera | |
| 81.94% | FJ459760 | 1603 | *Stenophora robusta* | Eugregarinorida | *Oxidus gracilis* | Diplopoda | |
| 80.93% | FJ459750 | 1768 | *Hoplorhynchus acanthatholius* | Eugregarinorida | *Enallagma civile* | Odonata | |
| 81.21% | FJ459756 | 1703 | *Prismatospora evansi* | Eugregarinorida | *Anax junius* | Odonata | |
| 81.15% | FJ459755 | 1698 | *Paraschneideria metamorphosa* | Eugregarinorida | *Sciara coprophila* | Diplopoda | |
| 79.45% | FJ459739 | 1592 | *Geneiorhynchus manifestus* | Eugregarinorida | *Anax junius* | Odonata | |
| 79.45% | FJ459738 | 1634 | *Colepismatophila watsonae* | Eugregarinorida | *Lepisma saccharina* | Thysanuron | |
| 81.47% | FJ459762 | 1697 | *Xiphocephalus ellisi* | Eugregarinorida | *Eleodes opaca* | Coleoptera | |
| 80.39% | FJ459761 | 1730 | *Stylocephalus giganteus* | Eugregarinorida | *Eleodes obscura* | Coleoptera | |
| 81.44% | FJ459763 | 1687 | *Xiphocephalus triplogemmatus* | Eugregarinorida | *Eleodes tricostata* | Coleoptera | |
| 79.49% | AF286023.1 | 1682 | *Hematodinium sp.* MF-2000 | Hematodinium | Unknown (Crab) | Unknown | |
